# Design and Validation of an Instrument for the Evaluation of the Quality of Mother-Child Bond and Attachment: “Cuestionario Vínculo y Apego Materno-Filial” (VAMF Questionnaire)

**DOI:** 10.1155/2024/6384511

**Published:** 2024-08-07

**Authors:** Maria Antonia Diaz-Ogallar, Antonio Hernandez-Martinez, Manuel Linares-Abad, Juan Miguel Martinez-Galiano

**Affiliations:** ^1^ Unit of Clinical Management Jodar Andalusian Health Service 23500, Jodar, Spain; ^2^ Nursing Department University of Jaen 23071, Jaen, Spain; ^3^ Department of Nursing Physiotherapy and Occupational Therapy Ciudad Real Faculty of Nursing University of Castilla-La Mancha 13071, Ciudad Real, Spain; ^4^ Consortium for Biomedical Research in Epidemiology and Public Health (CIBERESP) 28029, Madrid, Spain

## Abstract

The relationship between a mother and her newborn can be determined through two concepts: “bond” and “attachment.” Currently, there are no instruments that assess these phenomena jointly. *Objective*. This study aims to develop a valid and reliable instrument to determine the quality of the postnatal bond and attachment in the mother-child relationship. *Methods*. In Spain, a multidisciplinary panel of experts was involved in creating the “Maternal-Child Bond and Attachment (VAMF, for its initials in Spanish)” tool. The tool was piloted on a group of women and applied to the target population of women with children aged between 6 weeks and 18 months to determine the psychometric characteristics: internal consistency Cronbach's *α* was used. An exploratory factor analysis was conducted, a study of convergent validity with the scale and predictive validity with the Maternal Postnatal Attachment Scale (MPAS) using Pearson's correlation coefficient, and a study of reliability was carried out using the intraclass correlation coefficient. *Results*. 1155 women participated, with a mean age of 34.5 years. The VAMF showed good internal consistency for the scale with 29 items (*α* = 0.836). In the exploratory factor analysis (EFA), an explained variance of 49.71% was observed with 6 components. Convergent validity showed an adequate correlation, with a Pearson correlation coefficient with the MPAS scale of 0.679. The VAMF questionnaire presented an excellent predictive capacity in the bond subscale, with an area under the ROC curve of 0.90 (95% CI: 0.87–0.93), and a poor predictive capacity in the attachment subscale, with an area under the ROC curve of 0.69 (95% CI: 0.63–0.76) to predict MPAS scale scores. In the test-retest, the VAMF presented a very good adequate degree of absolute agreement (ICC: 0.86; 95% CI: 0.72–0.93). *Conclusions*. The VAMF is a new valid and reliable instrument for determining the quality of the mother-child bond and attachment.

## 1. Introduction

The development of the mother-newborn relationship is crucial, as the way in which this relationship is established will determine its future evolution, and creates the precedent on which the rest of the newborn relationships will be established throughout life [1, 2].

Different factors influence the formation of a mother-child relationship and can affect bonding and attachment, key aspects within this relationship, and can cause issues in both the mother and the newborn. Maternal mental health influences the formation of the maternal-child bond, and the presence of depressive symptoms, anxiety, or posttraumatic stress symptoms are related to difficulties in establishing this bond and adapting to the maternal role, difficulties in the response of the mother toward the needs of the newborn, and issues in the level and quality of subsequent attachment [3–8]. Likewise, the health status of the newborn, prematurity, and the presence of comorbidities are factors that affect the bonding process and the establishment of attachment [8]. High levels of stress hinder the development of a bond, change the quality of the interaction between the mother and child, and affect the mother's behavior, which may, in turn, affect the child's behavior and executive function, which is responsible for monitoring and regulating cognitive processes during the performance of complex cognitive tasks [8–10]. Adequate quality in mother-child interaction facilitates the child's socioemotional, behavioral, and cognitive development and could even be related to the child's physical health [11]. Alhusen et al. [12] highlighted the importance of the mother-child bond as a health concept, considering it a predictor of neonatal health and well-being.

Most women establish an adequate relationship with their children, although a significant number of mothers present difficulties in establishing this relationship [1]. In the United States, it is estimated that approximately 65–70% of children have a secure attachment style, while the remaining 30% have an insecure attachment style [13].

Two fundamental concepts are related to the relationship between mother and child: “bond” and “attachment.” These two concepts are closely related and are even used as synonyms to refer to the mother-child relationship, but both cover different aspects and moments of the said relationship [14–17].

The term “attachment” is a broad and complex concept within the mother-child relationship. Attachment can be considered as the affective bond that an individual, the newborn, establishes with a specific figure, in this case, the mother, and is a discriminatory, specific, and lasting behavior [18]. Attachment refers to reciprocity and interaction between the mother and child [15]. It can be understood as an affective bond of social origin, not innate, based on cognitive, emotional, and behavioral components and remains relatively stable throughout life. It manifests itself in the form of efforts by the newborn to seek and maintain proximity to the mother, behaviors encompassed within the “attachment behavior” [19, 20]. Hence, establishing attachment relationships with others is fundamental for human survival [21].

Three types of attachments have been established. Children with a secure attachment style use their mothers as a secure base for exploration, intensifying attachment behavior in episodes of separation. When reuniting with the mother, the child seeks contact and proximity. Children with an insecure-avoidant attachment style are quite independent and engage in exploratory behavior regardless of the mother's presence. When they are separated from their mother, they barely cry; when reunited, they mix proximity-seeking and avoidance behaviors. Finally, children with an insecure-ambivalent attachment style show signs of anxiety and distress during separation. When the mother returns, they seek close contact mixed with anger and resistance [13, 21–23].

A bond is understood as the emotional connection or union that a mother experiences toward her child [15, 16, 24]. It could be defined as a maternal affective state, with the mother's feelings and emotions toward her child as indicators of the bond established between the two [24]. This bond begins to form during pregnancy, and even before it, and continues to develop after birth [17, 25].

The bond is unidirectional, from the mother to the fetus, referring to the mother's emotional response to her child. In contrast, attachment is bidirectional, between the mother and the newborn, specifically referring to the reaction of the newborn, in the form of organized and stable behavior directed toward the main caregiver, the mother, in order to ensure the protection and safety of the newborn [15–17, 26–28]. The bond develops in the early phase of the maternity process and is related to the care provided by the mother to the newborn; while attachment is a later process, which generally occurs after childbirth, and is focused on the search for care by the newborn [14, 16, 26, 27].

The use of both concepts to refer to the mother-child relationship is complex, which is why some authors choose to use the term “mother-child relationship,” which combines both “bond” and “attachment” [15, 16, 29].

There are instruments designed to measure bond and instruments that measure attachment, with the following standing out: Maternal-Fetal Attachment Scale (MFAS) [15, 30], Prenatal Attachment Inventory (PAI) [15, 31], Maternal Antenatal Attachment Scale (MAAS) [15, 32], Pre- and Postnatal Bonding Scale (PPBS) [15, 33], Maternal Postnatal Attachment Scale (MPAS) [15, 34], Postpartum Bonding Questionnaire (PBQ) [15, 35–37], and y Maternal-to-Infant Bonding Scale (MIBS) [15, 38].

In addition, there are other measurement instruments focused on mother-child interaction in specific situations, particularly evaluating the child's reaction through a series of objective parameters, in order to determine the type of attachment. Among these instruments are the aforementioned “stranger situation” and the Massie–Campbell scale [39].

Different measurement instruments assess the quality of the bond, pre- and/or postnatal, and the attachment between mothers and children. These instruments focus on determining either the bond or attachment, closely related phenomena that are the cornerstone of the mother-child relationship. However, no single instrument has been identified that allows the assessment of both parameters, either to assess the joint impact of both parameters or independently, depending on the interest at each moment with the advantages that this entails. There is a need for an instrument that can determine the quality of bonding and attachment using a single questionnaire, in order to early detect suboptimal bonding and/or attachment in newborns aged between 6 weeks and 18 months. This instrument could be used in a quick, easy, and practical way in clinical-care settings to intervene and prevent early dysfunctions that may have negative consequences for the mother-child dyad.

Therefore, we aimed to develop a valid and reliable instrument to determine the quality of the postnatal bond and attachment in the mother-child relationship.

## 2. Methods

### 2.1. Phase 1: Design and Questionnaire Development

A bibliographic search was carried out in the main health sciences databases (Scopus, Web of Science (WoS), ProQuest, CINAHL, and PubMed) from November 2021 to February 2022.

After reviewing in detail 40 existing instruments for measuring the bond, attachment, and different aspects of the mother-child relationship, and after consulting with professionals in pediatrics, neonatology, midwifery, nursing, psychology, psychiatry, and anthropology, a questionnaire was developed comprising of 31 items divided into two subscales: a bond subscale made up of 18 items and an attachment subscale made up of 13 items. This led to version 0 of the questionnaire “Mother-Child Bond and Attachment” (VAMF, *for its initials in Spanish*).

### 2.2. Phase 2: Expert Panel

After obtaining version 0 of the VAMF questionnaire, it was evaluated by a multidisciplinary panel of experts. Twelve experts from different disciplines were contacted: nursing, pediatrics, midwifery, pediatric nursing, psychiatry, neonatology, psychology, and anthropology, for its evaluation. It was also taken into account that the experts came from different regions of Spain to consider the cultural and linguistic variety that could exist when interpreting the questions and answers of the questionnaire. They were contacted via e-mail, inviting them to participate in this research as experts in mother-child relationships by evaluating the questionnaire prepared. Once they agreed to participate, they were sent the original questionnaire, and they were asked to evaluate it, assigning a score of 1 (best score) to 5 (worst score) to each of the items that make up the questionnaire based on four parameters: writing, understanding, relevance and general assessment. Likewise, a section was included for them to provide any observations they considered appropriate on each item. Regarding the evaluation of the questionnaire in general, an observation section was set up for its evaluation in general terms and for them to make the contributions/suggestions they considered appropriate.

Once the 12 evaluations were received, the feedback was collated, and the appropriate corrections were made based on the experts' opinions, obtaining version 1 of the questionnaire. The modified questionnaire was sent back to the panel of experts for a second evaluation, after which their approval was received.

### 2.3. Phase 3: Pilot of Questionnaire

Women with children between the ages of 6 weeks and 18 months were actively recruited in the Nursing (through the Healthy Child Program and the Vaccination Program), Midwife, and Pediatrics offices of the Dr. Ricardo Fernández Valadés Health Center (Jódar, Jaén) between the months of May and August 2022. A sample of 15 mothers was obtained who were given appointments to come in and complete the VAMF questionnaire together with the MPAS questionnaire. Once the questionnaire was completed, an interview was conducted with each mother so that they could evaluate the VAMF and make the contributions they considered appropriate: they assessed whether the statement of each item was correct, understandable, and had an adequate length; the adequacy of the items; the categorization of the answers; the general opinion of the mother about the items; length; and format of the questionnaire and if they found any difficulty when completing it. In addition, they were asked to evaluate the questionnaire in general and to make any contributions or suggestions they considered appropriate.

After this pilot, one of the items was modified, obtaining version 2 of the questionnaire.

### 2.4. Phase 4: Application of the Instrument to the Target Population to Determine Its Psychometric Properties

#### 2.4.1. Design and Subject Selection

For this part, a cross-sectional validation study was carried out with women who had a child in Spain. This phase of the study was carried out from September 2022 to March 2023 in Spain. The inclusion criteria were women aged between 18 and 45 years, with a biological child whose age is between 6 weeks and 18 months, who have previously signed the informed consent for participation in the study. The exclusion criteria were mothers of multiple births (two or more newborns) and those who do not speak or do not know the Spanish language (language barrier).

To recruit women for our study sample, different associations that were related to pregnancy, childbirth, and postpartum and support groups for breastfeeding and parenting throughout the Spanish territory were contacted. Following the application of inclusion and exclusion criteria, participants were informed about the objective and mode of participation in the study. Once they consented to participate, they were provided with the questionnaire online.

The sample size was estimated according to the criteria for carrying out a factorial analysis. These criteria consider 10 subjects for each item [40]. Therefore, we needed a sample of at least 310 participants.

#### 2.4.2. Information Sources

To collect the necessary information for validation, a questionnaire was developed consisting of sociodemographic and clinical variables of both the mother and the newborn, which included the validated tool “MPAS” [34, 41], and the new tool to be validated, “VAMF” version 2. For its distribution, contact was made with different associations related to pregnancy, childbirth, and the postpartum period, and with support groups for breastfeeding and parenting throughout the Spanish territory. After applying the inclusion and exclusion criteria, the participating women were informed and accepted the informed consent for participation in the research, and the questionnaire was administered to them.

#### 2.4.3. Measuring Instruments

The postnatal attachment was evaluated using the “Maternal Postnatal Attachment Scale” (MPAS) questionnaire. This questionnaire was developed by Condon and Corkindale in 1998 and assesses the mother's emotional response to her newborn. It is made up of 19 items that describe the mother's feelings toward her baby/child. Each item is scored using a Likert-type scale ranging from 1 (worst score) to 5 (highest score), except for items with inverse scores. The lower the score, the less the bond between the mother and the child. The MPAS presents good psychometric capabilities, with adequate internal consistency (*α* = 0.78) [34, 38, 41].

### 2.5. Statistical Analysis

For sociodemographic and clinical data, absolute and relative frequencies were used to describe the qualitative variables, and the mean and standard deviation (SD) were used to describe the quantitative variables.

First, the reliability analysis was performed using Cronbach's *α* to assess internal consistency (IC). The IC tells us to what extent the items in question are correlated with each other and how they fit together and measure the same concept. Cronbach's *α* is one of the most widely used measures to assess the reliability of a scale. Its values range from 0 to 1. One of the most accepted rules is to consider *α* > 0.9 as excellent, *α* > 0.8 as good, *α* > 0.7 as acceptable, *α* > 0.6 as questionable, *α* > 0.5 as poor, and *α* < 0.5 as unacceptable [42].

To determine the scale's validity, we analyzed three of the most common types: construct validity, convergent validity, and criterion validity.

Regarding construct validity, we carried out an exploratory factor analysis (EFA) to determine the underlying factors through the principal component analysis (PCA). Before performing the EFA, we analyzed the Kaiser–Meyer–Olkin (KMO) tests and Bartlett's tests of sphericity, which indicate whether this analysis was appropriate to apply. For this to be the case, the KMO must be above 0.6 and as close as possible to 1, and the Bartlett sphericity, which consists of the statistical hypothesis test, must be less than 0.05 to reject the null hypothesis of sphericity and ensure that the factorial model is adequate to explain the data. In the EFA, we use varimax rotation to help clarify the allocation of items to different factors. To determine the number of factors to maintain, we used the Kaiser criterion, which is one of the most widely used criteria; factors with eigenvalues greater than unit value are retained [43].

Next, the convergent validity was studied. For this, we studied the relationship between the VAMF and the MPAS using the Pearson correlation coefficient, as well as the predictive capacity of the VAMF scale and its two attachment and bond subscales with the MPAS scale. In addition, the predictive capacity of the VAMF scale over the MPAS scale was estimated. To do so, the MPAS variable was dichotomized for a score below the 10th percentile (low bond). The area under the ROC curve (AUC) was estimated with their respective 95% confidence intervals, as well as the Youden index to determine the best cut-off point.

The next step was to determine the criterion validity. In this analysis, the scores obtained in the test were compared with some factors that could be an obstacle to the establishment of the bond and secure attachment, such as the type of birth, admission of the newborn, type of breastfeeding, hospital stay, and skin-to-skin contact. Thus, a bivariate analysis was carried out using the Pearson or Student–Fisher chi-square tests, depending on the qualitative or quantitative nature of the variables. The results were considered statistically significant when *p* ≤ 0.05.

Lastly, intraobserver reliability was evaluated using a test-retest test in a sample of 30 women who took the questionnaire again 24 hours after completing the first one, using the intraclass correlation coefficient in this case.

Version 24.0 of the statistical package SPSS was used.

### 2.6. Ethical Considerations

The Research Ethics Committee of the Province of Jaen approved this study (DCVA-21/2012-N-21). First, mothers had to read the information sheet about the study and its objectives and then they accepted the informed consent for participation in the study. Once the informed consent was accepted, they completed the questionnaire.

All methods were carried out in accordance with relevant guidelines and regulations. Informed consent was obtained from all participants.

## 3. Results

### 3.1. Participant Characteristics

1155 women participated (66 declined to participate) with a mean age of 34.5 years (SD = 3.90), and 58.4% (675) were married. 88% (1016) did not have any disease. For 56.5% (653) of the women, this was their first pregnancy. 41.9% (484) reported having had health problems related to pregnancy. 58.6% (667) of the women had a normal vaginal birth, and 80% (924) used analgesia during it. 78.4% (905) started breastfeeding early after birth, and 83.7% (967) had skin-to-skin contact after birth. Regarding the babies, the mean age was 8.6 (SD = 5.56) months, 4.6% (53) were premature, and 83.5% (965) were still breastfeeding. The data can be consulted in Table 1.

### 3.2. Psychometric Properties

#### 3.2.1. Internal Consistency

To assess the internal consistency, Cronbach's *α* was used for the entire questionnaire. During the first evaluation of all the items, it was observed that removing items 5 and 6 improved the scale's total score. Thus, for the scale without these two items, the *α* was 0.836. With this modification, all alpha values scored above 0.823 when removing an item. The *α* values for each factor are shown in Table 2. After removing these items, we proceeded with a new enumeration of the questions, modifying the scale to 29 items (version 3 of the questionnaire). All corrected item-total correlations were above 0.

#### 3.2.2. Intraobserver Reliability (Intraobserver Stability)

Finally, the test-retest test was carried out by administering the VAMF questionnaire to a group of 30 mothers who completed it twice, separated by a period of 24 hours. The analysis showed excellent agreement, with an intraclass correlation coefficient of absolute agreement of 0.86 (95% CI: 0.72–0.93) and a consistency intraclass correlation coefficient of 0.85 (95% CI: 0.71–0.93).

#### 3.2.3. Exploratory Factor Analysis

The KMO test gave a value of 0.901, and the Bartlett test of sphericity was <0.001. Therefore, the EFA was carried out. Six components explained 49.71% of the variance. The first component consisted of items 18, 19, 20, 22, 24, 25, 26, 27, and 29 and represented 21.14% of the variance. The second component, made up of items 3, 5, 6, 12, 14, 15, and 16, explained 11.04% of the total variance. The third factor, made up of items 1, 2, 7, 9, 13, and 17, explained 6.09% of the variance. The fourth factor, made up of items 10, 21, and 23, represented 4.31% of the variance. The fifth factor, which includes items 8 and 28, explained 3.62% of the variance. The sixth factor, which includes items 4 and 11, explained 3.52% of the variance. In addition, all anti-image diagonal correlations showed figures greater than 0.750, except for items 23 and 25 with values of 0.659 and 0.660, respectively.  Table 3 presents the items of the scale along with their respective factorial weights.

#### 3.2.4. Convergent Validity

Next, convergent validity was studied with the MPAS scale, observing a statistically significant association (*p* < 0.001) with a Pearson correlation coefficient with a total scale of 0.679. This correlation was also studied when separating the items related to the bond and to the attachment, observing for the case of the bond, a higher correlation coefficient of 0.766 and a lower attachment of 0.370.

#### 3.2.5. Predictive Validity

**Figure 1:**
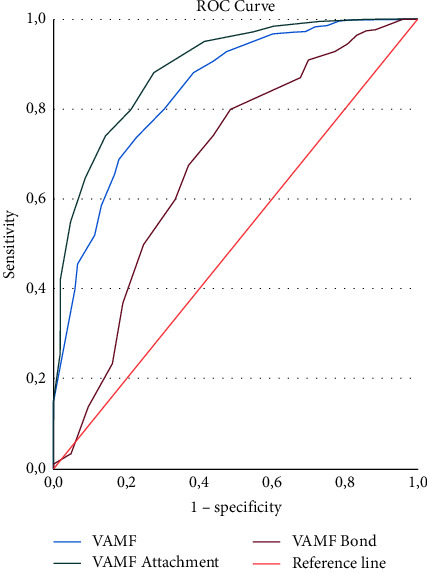
Predictive capacity of the VAMF questionnaire and the attachment and bond subscales for the MPAS scale for a percentile score of <10 (75 points).

In the case of predictive validity, it was decided to use the MPAS variable and use scores below the 10th percentile of the scale as the cut-off point, standing at 75 points. In this way, we determined the predictive capacity of the VAMF scale and the two attachment and bond subscales on the probability of having low scores on the MPAS. Observing that the VAMF scale, in the bond subscale, has an excellent predictive capacity for the MPAS scale with an area under the ROC curve of 0.90 (95% CI: 0.87–0.93), for the global VAMF scale, the predictive capacity was good with AUC of ROC of 0.85 (95% CI: 0.81–0.90), while the predictive capacity was poor if we used the attachment subscale with an AUC of ROC of 0.69 (95% CI: 0.63–0.76) (Figure 1). The Youden index was 98.5 points for the VAMF scale, 54.5 points for the VAMF-attachment subscale, and 40.5 points for its VAMF-attachment subscale.

#### 3.2.6. Criterion Validity

For this analysis, we studied the relationship between various variables that could affect the condition of the mother and the newborn and their relationship with the scores of the VAMF scale and the two attachment and bond subscales. As can be seen in Table 4, among the variables that could affect the maternal condition, we found a statistically significant association (*p*  <  0.05) between the VAMF scale and the type of birth; postpartum complications; hospital admission days; health status in the 6 weeks postpartum; mental health problems; depression during pregnancy; tiredness or fatigue during pregnancy, childbirth, or postpartum; and anxiety during pregnancy, childbirth, or postpartum. Regarding the bond subscale, a statistically significant association (*p*  <  0.05) was found with the variables type of birth; postpartum complications; days of hospital admission; ICU admission; state of health in the 6 weeks postpartum; mental health problems; depression during pregnancy; tiredness or fatigue during pregnancy, birth, and the postpartum period; and anxiety during pregnancy, birth, and the postpartum period. For its part, the score on the attachment subscale had a statistically significant association (*p*  <  0.05) with the type of birth variable. In Table 5, the variables related to the newborn that have a statistically significant association (*p*  <  0.05) with the total score of the VAMF scale were gestational age at the time of birth, hospital admission at birth, early initiation of breastfeeding, excessive crying, and colic of the infant. The bond subscale presented a statistically significant association (*p*  <  0.05) with the variables hospital admission at birth, health problems, excessive crying, and infant colic. In contrast, the score on the attachment subscale showed a statistically significant association with the early onset of breastfeeding and colic of the infant. The skin-to-skin contact variable did not present a statistically significant association with the VAMF scale (*p*=0.233) nor with the bond (*p*=0.114) and attachment (*p*=0.722) subscales.

## 4. Discussion

The VAMF questionnaire showed good internal consistency for the scale with 29 items. The EFA indicated that 49.71% of the variance of the VAMF was explained by dividing the items into 6 components. Convergent validity was studied with the MPAS scale [41], showing adequate correlation. Regarding predictive validity, the VAMF scale presented an excellent predictive capacity in the bond subscale and a poor predictive capacity in the attachment subscale. In the test-retest test, the VAMF showed good intraclass correlation.

The VAMF comprises two subscales, one for assessing the bond and the other for assessing the attachment. The total internal consistency of the VAMF was good (*α* = 0.836), higher than that identified in the original version of the MPAS questionnaire [34], which measures bonds after birth, and showed an acceptable internal consistency with a Cronbach's *α* of 0.78. Also, the Spanish version of the MPAS adapted in 2016 by Riera-Martín et al. [41] presented an acceptable internal consistency, with a Cronbach's *α* of 0.75, lower than that found in our results. The PBQ [35], used to measure the postpartum bond, presented good internal consistency, with Cronbach's *α* ranging from 0.76 to 0.87 on the global scale, and the scores are in line with those identified in the VAMF. However, the Spanish version of the PBQ, adapted and validated by García-Esteve et al. [44], obtained excellent internal consistency, with a Cronbach's *α* of 0.90 for the scale's total score, which is somewhat higher than that of the VAMF. In the convergent validity, compared to the MPAS scale, the VAMF presented a Pearson correlation coefficient of 0.679 for the total scale, showing a higher correlation in the bond subscale (*r* = 0.766) and lower for the attachment (*r* = 0.370). This correlation is in line with what each subscale measures, as the MPAS scale measures postnatal bonding, presenting a high correlation with the bonding subscale of the VAMF. In contrast, the attachment subscale measures a different phenomenon, with a poor correlation with the MPAS.

The analysis of the predictive validity showed an excellent predictive capacity in the bond subscale, while in the attachment subscale, it showed a poor predictive capacity, and these findings are in line with what was previously indicated. When using the MPAS scale for the analysis, as an instrument for measuring the postnatal bond with good psychometric capacities, it is seen that the predictive capacity of the questionnaire in the bond subscale is high, while the attachment subscale, which measures a reality closely related but different to the bond, shows a poor predictive ability. The predictive power of the global questionnaire is good. The PBQ questionnaire shows a positive predictive capacity of 0.79, varying with the presence and type of disorder in the relationship between mother and child, decreasing with the presence of rejection toward the child or maternal anger [35], and being poorer than that of the global VAMF questionnaire and much lower than that of the attachment subscale.

Regarding the maternal factors that are related to the scores of the VAMF questionnaire and its subscales, postpartum complications; hospital admission days; health status during the 6 weeks postpartum; mental health problems; depression during pregnancy; tiredness or fatigue during pregnancy, childbirth, and postpartum; and anxiety during pregnancy, childbirth, and postpartum had a statistically significant association with the VAMF global score. The bond subscale is related to the type of birth; postpartum complications; length of hospital admission; ICU admission; state of health in the 6 weeks postpartum; mental health problems; depression during pregnancy; tiredness or fatigue during pregnancy, birth, and postpartum period; and anxiety during pregnancy, birth, and postpartum. In the adaptation and validation of the Spanish version of the PBQ, a positive correlation was found between the presence of depressive symptoms and changes in the bond [44]. Likewise, Taylor et al. [38] found a relationship between depressive symptoms and mother-child bonding using the MIBS scales and the EDPS questionnaire, along with two other scales, showing that the presence of depression is related to worse bonding, which is in line with what was identified in our results. Likewise, the type of birth showed a correlation with the attachment subscale, which could be related to the increase in maternal oxytocin levels during childbirth, which could be a facilitator of mother-child attachment, related to the appearance of behaviors that favor bonding and maternal-filial attachment after childbirth, as well as with the increase in cortisol levels [45]. In the newborn, there is a release of norepinephrine, cortisol, and vasopressin during the passage through the birth canal, where the increase in the concentration of adrenaline would be related to the formation of attachment, since these levels are related to olfactory learning after the birth [45]. Likewise, maternal feelings before childbirth and the possible consequences for the newborn play a fundamental role in establishing attachment [8]. On the other hand, newborn factors such as the gestational age at the time of birth, hospital admission at birth, early initiation of breastfeeding, excessive crying, and infant colic showed an association with the overall VAMF score. Focusing on both subscales, the bond subscale is associated with hospital admission at birth, newborn health problems, excessive crying, and infant colic, while the attachment subscale is associated with early onset of breastfeeding and infant colic. Skin-to-skin contact did not correlate with the VAMF scale or with any of its two subscales. After conducting a review of the literature, Olza-Fernández et al. [45] showed the benefits of skin-to-skin contact, being an essential part in the development of the mother-child relationship, since it helps the newborn in conserving energy, adjusting the acid-base balance, regulates respiration, and has a calming effect, as well as increases maternal attention to the newborn and reduces maternal cortisol levels. During skin-to-skin contact, an oxytocin discharge is produced, which is related to the increased response of mothers to the signals emitted by the newborn and could play an important role in the initiation of breastfeeding [45]. Klaus and Kennell [46] in their “bonding theory,” affirmed that skin-to-skin contact was the determining factor for the development of the bond between the mother and the child. However, as has also been identified in our results, other authors, such as Myers [47], indicated that, while the premise that skin-to-skin contact is a determining factor is true, it is not the only one related to bond development.

### 4.1. Strengths and Limitations

The number of participants in this study was high, much greater than the minimum sample size calculated. The sample includes women from different regions of Spain, which is why possible linguistic, social, or cultural differences that may exist between these areas may be reflected. As it is a questionnaire, a possible selection bias associated with nonresponse must not be ruled out because the number of women who did not respond was very low. Although, there are no indications or reasons to suggest that the women who did not participate would have responded differently from those who did. The group of experts was made up of the different professional profiles that are involved in the attachment, bonding, and parenting process. These experts have extensive experience in teaching, clinical care, and research on the parenting, attachment, and bonding process. The authors have already validated and designed instruments that are applied in the perinatal stage [48].

## 5. Conclusions

In conclusion, the VAMF is a new tool that presents good psychometric properties for measuring the mother-child bond and attachment in mothers and children aged between 6 weeks and 18 months and can be applied easily by professionals in the clinical-care setting. Due to its good psychometric capabilities, this tool could be systematically integrated into clinical practice in pediatric or pediatric nursing consultations. It could be employed as a routine component of the health assessment for a child, aimed at identifying any disruptions in the mother-child relationship between 6 weeks and 18 months of age. This approach would function as a screening mechanism, assisting healthcare professionals in early detection and enabling targeted interventions to strengthen the bond and attachment.

### 5.1. Relevance for Clinical Practice

The creation of the VAMF questionnaire will allow us to detect bond and attachment alterations in an easy and quick way by nurses and doctors in contact with mothers and children in the clinical practice. Early detection of suboptimal forms of bond and/or attachment will help medical staff to prevent negative consequences on mother-child dyads, such as mental health problems on mothers or difficulties in establishing new relationships in the future by the child. A future line of continuity with the topic is proposed in which the questionnaire is applied and the consequences and factors that are associated with the quality of the maternal-child bond and attachment are known.

## Figures and Tables

**Table 1:** Descriptive characteristics of the study participants.

Variable	*N* = 1155 *N* (%)
Maternal age mean (SD)	34.5 (3.90)

*Civil status*	
Married	675 (58.4)
Common-law couple	184 (15.9)
Single	284 (24.6)
Divorced	9 (0.8)
Separated	2 (0.2)
Widowed	1 (0.1)

*Nationality*	
Spanish	1127 (97.6)
Not Spanish	28 (2.4)

*Approximate income level*	
<1000 euros/month	221 (19.1)
Between 1000 and 1999 euros/month	618 (53.5)
Between 2000 and 2999 euros/month	255 (22.1)
>3000 euros/month	61 (5.3)

*Current illness*	
No	1016 (88.0)
Yes	139 (12.0)

*Number of pregnancies*	
1	653 (56.6)
2	326 (28.2)
≥3	174 (15.2)

*Number of miscarriages*	
0	799 (69.2)
1	263 (22.8)
2	66 (5.7)
≥3	27 (2.3)

*Number of vaginal births*	
None	240 (20.8)
One	687 (59.5)
Two	201 (17.4)
Three or more	27 (2.3)

*Number of cesareans*	
None	850 (73.6)
One	272 (23.5)
Two	29 (2.5)
Three or more	4 (0.4)

*Number of children*	
None	3 (0.3)
One	844 (73.1)
Two	271 (23.5)
Three or more	37 (3.1)

*High-risk pregnancy*	
No	954 (82.6)
Yes	186 (16.1)
NA	15 (1.3)

*Planned pregnancy*	
No	109 (9.4)
Yes	1046 (90.6)

*Reproductive technique*	
No	991 (85.8)
Yes	164 (14.2)

*Attended antenatal classes*	
No	351 (30.4)
Yes	804 (69.6)

*Type of birth*	
Normal	677 (58.6)
Instrumental	219 (19.0)
Scheduled cesarean	68 (5.9)
Emergency cesarean	191 (16.5)

*Analgesia/anesthesia*	
No	231 (20.0)
Yes	924 (80.0)

*Weeks at birth*	
Term (≥37 weeks)	1096 (94.9)
Moderate preterm (32–37 weeks)	50 (4.3)
Very preterm (28–32 weeks)	7 (0.6)
Extreme preterm (<28 weeks)	2 (0.2)

*Problems during pregnancy*	
No	671 (58.1)
Yes	484 (41.9)

*Early initiation of breastfeeding*	
No	235 (20.3)
Yes	905 (78.4)
NA	15 (1.3)

*Skin-to-skin contact*	
No	185 (16.0)
Yes	967 (83.7)
NA	3 (0.3)

*Complications after birth*	
No	963 (83.4)
Yes	192 (16.6)

*Length of hospital stay*	
1 day	144 (12.5)
Two days	605 (52.4)
Three days	273 (23.6)
Between 3 days and one week	123 (10.6)
More than one week	10 (0.9)

*ICU admission*	
No	1138 (98.5)
Yes	17 (1.5)

*Hospital readmission*	
No	1129 (97.7)
Yes	26 (2.3)

*Mental health illness*	
No	872 (75.5)
Yes	283 (24.5)

*Smoker*	
No	1046 (90.6)
Yes	109 (9.4)

*Frequency of alcohol consumption*	
None	379 (32.8)
Occasional	694 (60.1)
Habitual	82 (7.1)

Age of newborn (SD)	8.6 (5.56)

*Sex of newborn*	
Female	620 (54.1)
Male	527 (45.9)

*Maintenance of breastfeeding*	
No	190 (16.5)
Yes	965 (83.5)

NA, not available.

**Table 2:** Internal consistency VAMF (version 3) after eliminating 2 items.

Variable	Corrected item-total correlation	Cronbach's *α*
Total		0.836

*On removing items*		
(1) I feel happy when I am with my baby	0.461	0.827
(2) I feel overwhelmed when I am with my baby	0.262	0.833
(3) I like rocking or hugging my baby	0.293	0.833
(4) I feel proud when my baby does new things	0.157	0.836
(5) I like the time I spend with my baby	0.487	0.827
(6) I like to play with my baby	0.370	0.830
(7) I feel calm when I am with my baby	0.490	0.826
(8) Seeing my baby smile makes me happy	0.163	0.836
(9) My baby's cry irritates or annoys me or makes me feel helpless	0.274	0.835
(10) I find it difficult to separate from my baby	0.251	0.837
(11) I feel capable of identifying my baby's needs	0.258	0.835
(12) I like to talk to my baby	0.350	0.832
(13) Being with my baby makes me anxious and/or stressed	0.340	0.831
(14) I feel like I love my baby a lot	0.392	0.832
(15) Looking after my baby gives me satisfaction	0.474	0.827
(16) I don't feel caring with my baby	0.330	0.831
(17) My baby is calm when with me	0.526	0.825
(18) My baby smiles spontaneously	0.520	0.825
(19) My baby laughs when I play with them	0.528	0.825
(20) My baby maintains their gaze at me	0.477	0.827
(21) My baby is irritable or restless when not with me	0.110	0.840
(22) My baby perceives my feelings via my gestures and tone of voice	0.396	0.829
(23) My baby cries when I am no longer present	0.163	0.838
(24) My baby shows signs of happiness when seeing me again after a period of separation	0.503	0.824
(25) My baby calms when hearing my voice	0.543	0.823
(26) When I smile at my baby, my baby smiles back	0.502	0.825
(27) My baby tries to get my attention frequently	0.287	0.834
(28) My baby prefers to be in the crib or pram instead of my arms	0.104	0.837
(29) My baby expresses happiness at my displays of affection (hugs, kisses, etc.)	0.530	0.824

**Table 3:** Rotated component matrix.

Item	Components					
	1	2	3	4	5	6
(1) I feel happy when I am with my baby	0.101	0.477	**0.532**	0.032	0.044	−0.041
(2) I feel overwhelmed when I am with my baby	−0.038	0.144	**0.672**	−0.015	0.012	−0.117
(3) I like rocking or hugging my baby	0.044	**0.623**	0.046	0.038	−0.045	−0.075
(4) I feel proud when my baby does new things	0.029	0.058	−0.067	0.066	0.357	**0.730**
(5) I like the time I spend with my baby	0.075	**0.612**	0.444	0.050	−0.044	0.089
(6) I like to play with my baby	0.074	**0.644**	0.181	−0.015	0.006	0.098
(7) I feel calm when I am with my baby	0.181	0.340	**0.596**	0.003	−0.003	0.157
(8) Seeing my baby smile makes me happy	0.042	0.259	−0.005	−0.048	**0.304**	0.080
(9) My baby's cry irritates or annoys me or makes me feel helpless	0.107	0.013	**0.618**	−0.065	−0.128	0.068
(10) I find it difficult to separate from my baby	0.027	0.304	0.071	**0.487**	−0.079	0.049
(11) I feel capable of identifying my baby's needs	0.188	0.097	0.155	−0.009	−0.226	**0.609**
(12) I like to talk to my baby	0.114	**0.614**	0.023	0.016	0.008	0.139
(13) Being with my baby makes me anxious and/or stressed	0.020	0.230	**0.703**	−0.105	0.189	−0.003
(14) I feel like I love my baby a lot	0.097	**0.651**	0.132	0.030	0.173	−0.032
(15) Looking after my baby gives me satisfaction	0.102	**0.571**	0.377	0.033	0.068	0.134
(16) I don't feel caring with my baby	0.144	**0.390**	0.252	0.029	0.167	−0.165
(17) My baby is calm when with me	0.419	0.155	**0.475**	0.071	0.013	0.201
(18) My baby smiles spontaneously	**0.791**	0.079	0.032	−0.050	0.032	0.037
(19) My baby laughs when I play with them	**0.793**	0.076	0.077	−0.105	0.030	0.045
(20) My baby maintains their gaze at me	**0.688**	0.135	0.021	−0.004	0.084	−0.021
(21) My baby is irritable or restless when not with me	0.027	−0.051	−0.047	**0.755**	0.042	0.028
(22) My baby perceives my feelings via my gestures and tone of voice	**0.471**	0.046	0.095	0.272	−0.110	0.115
(23) My baby cries when I am no longer present	0.108	−0.056	−0.075	**0.785**	0.086	−0.010
(24) My baby shows signs of happiness when seeing me again after a period of separation	**0.673**	0.065	0.057	0.218	0.002	0.044
(25) My baby calms when hearing my voice	**0.513**	0.122	0.303	0.231	−0.124	0.128
(26) When I smile at my baby, my baby smiles back	**0.775**	0.047	0.031	−0.022	−0.003	0.038
(27) My baby tries to get my attention frequently	**0.469**	0.035	−0.166	0.416	0.070	−0.104
(28) My baby prefers to be in the crib or pram instead of my arms	0.044	0.024	0.049	0.103	**0.832**	−0.018
(29) My baby expresses happiness at my displays of affection (hugs, kisses, etc.)	**0.685**	0.106	0.136	0.037	0.083	0.027

The bold values indicate the highest correlation between the variable and the component, and shows in which component the item is located.

**Table 4:** Relationship between variables that affect the mother and the VAMF score, and the bond and connection subscales (criterion validity).

*N* = 1155	Attachment subscale		Bond subscale		VAMF scale	
Variables related to the maternal condition	Mean (SD)	*P* value	Mean (SD)	P value	Mean (SD)	*P* value
Type of birth		**0.019**		**0.002**		**0.018**
Normal	43.5 (4.41)		57.9 (3.64)		101.4 (6.49)	
Instrumental	42.7 (4.27)		58.0 (3.89)		100.8 (6.58)	
Scheduled cesarean	42.2 (4.74)		56.3 (3.89)		100.5 (7.31)	
Emergency cesarean	42.9 (5.19)		56.8 (4.99)		99.7 (8.60)	
Postpartum complications		**0.662**		**<0.001**		**0.019**
No	43.2 (4.59)		57.9 (3.86)		101.2 (6.89)	
Yes	43.1 (4.42)		56.8 (4.37)		99.9 (7.27)	
Length of hospital stay		**0.177**		**0.050**		**0.053**
One day	44.0 (4.02)		58.2 (3.49)		102.2 (6.19)	
Two days	43.3 (4.46)		57.8 (3.82)		101.1 (6.73)	
Three days	42.9 (4.73)		57.8 (4.06)		100.6 (6.96)	
Between 3 days and one week	43.0 (4.96)		56.9 (4.29)		99.9 (8.04)	
More than one week	42.3 (6.77)		56.1 (9.24)		98.4 (13.81)	
ICU admission		**0.505**		**0.037**		**0.453**
No	43.2 (4.56)		57.8 (3.89)		101.0 (6.90)	
Yes	43.9 (4.34)		55.8 (7.55)		99.7 (10.82)	
Health status during the 6 postpartum weeks		**0.996**		**<0.001**		**0.002**
Very bad	43.1 (4.78)		55.1 (6.96)		98.2 (10.70)	
Bad	43.1 (4.76)		56.8 (5.02)		99.9 (8.36)	
Ok	43.3 (4.52)		57.1 (4.17)		100.4 (7.30)	
Good	43.2 (4.51)		57.7 (3.52)		100.9 (6.40)	
Very good	43.2 (4.61)		58.8 (3.41)		102.0 (6.49)	
Mental health illness		**0.201**		**<0.001**		**<0.001**
No	43.3 (4.44)		58.1 (3.64)		101.4 (6.62)	
Yes	42.9 (4.91)		56.7 (4.71)		99.6 (7.81)	
Depression during pregnancy		**0.244**		**0.009**		**0.024**
No	43.3 (4.51)		57.9 (3.77)		101.1 (6.76)	
Yes	42.7 (4.99)		56.8 (5.46)		99.6 (8.61)	
Tiredness or fatigue during pregnancy, birth, and postpartum period		**0.898**		**<0.001**		**0.008**
No	43.2 (4.20)		59.4 (3.05)		102.5 (5.54)	
Yes	43.2 (4.60)		57.6 (4.03)		100.8 (7.10)	
Anxiety during pregnancy, birth, and postpartum period		**0.687**		**<0.001**		**<0.001**
No	43.3 (4.42)		58.7 (3.11)		102.0 (6.07)	
Yes	43.2 (4.70)		56.8 (4.49)		99.9 (7.66)	

The bold values should correspond with those that have reached statistical significance.

**Table 5:** Relationship between variables that affect the neonate and the VAMF score, and the bond and connection subscales (criterion validity).

*N* = 1155	Attachment subscale		Bond subscale		VAMF scale	
Variables related to the neonate	Mean (SD)	*P* value	Mean (SD)	P value	Mean (SD)	*P* value
Gestational weeks at the time of birth		**0.109**		**0.195**		**0.058**
Term (≥37 weeks)	43.3 (4.50)		57.8 (3.85)		101.1 (6.83)	
Moderate preterm (32–37 weeks)	41.7 (5.65)		56.6 (6.16)		98.4 (9.54)	
Very preterm (28–32 weeks)	44.6 (3.41)		57.4 (1.13)		102.0 (4.12)	
Extreme preterm (<28 weeks)	42.5 (3.54)		59.5 (3.54)		102.0 (7.07)	
Hospital admission at birth		**0.104**		**0.090**		**0.037**
No	43.3 (4.47)		57.9 (3.84)		101.2 (6.73)	
Yes, in the neonatal unit	42.3 (5.18)		57.2 (5.08)		99.5 (8.90)	
Yes, in the neonatal intensive care unit	42.9 (5.07)		56.8 (4.04)		99.7 (7.27)	
Health problems		**0.256**		**<0.001**		**0.211**
No	43.2 (4.55)		57.9 (3.80)		101.1 (6.88)	
Yes	43.7 (4.62)		56.5 (5.13)		100.2 (7.75)	
Early initiation of breastfeeding		**0.028**		**0.058**		**0.008**
No	42.6 (4.93)		57.2 (4.90)		99.8 (8.17)	
Yes	43.4 (4.45)		57.9 (3.69)		101.3 (6.59)	
NA	41.9 (4.45)		57.0 (3.68)		98.9 (7.24)	
Skin-to-skin contact		**0.722**		**0.114**		**0.233**
No	43.0 (5.15)		57.3 (5.18)		100.4 (8.97)	
Yes	43.3 (4.44)		57.9 (3.69)		101.1 (6.51)	
NA	41.7 (6.51)		55.0 (4.36)		96.7 (10.69)	
Excessive crying		**0.075**		**<0.001**		**<0.001**
No	43.3 (4.45)		58.0 (3.77)		101.3 (6.70)	
Yes	42.4 (5.62)		54.9 (5.05)		97.3 (8.84)	
Newborn colic		**<0.001**		**<0.001**		**<0.001**
No	43.7 (4.30)		58.1 (3.63)		101.8 (6.50)	
Yes	43.0 (4.35)		57.5 (4.16)		100.5 (6.83)	
NA	40.6 (5.98)		55.9 (5.02)		96.5 (8.72)	

NA, not available/not known. The bold values should correspond with those that have reached statistical significance.

## Data Availability

The data that support the findings of this study are available on request from the corresponding author. The data are not publicly available due to privacy or ethical restrictions.
